# Functional–Structural Plant Model “GreenLab”: A State-of-the-Art Review

**DOI:** 10.34133/plantphenomics.0118

**Published:** 2024-02-07

**Authors:** Xiujuan Wang, Jing Hua, Mengzhen Kang, Haoyu Wang, Philippe de Reffye

**Affiliations:** ^1^ State Key Laboratory of Multimodal Artificial Intelligence Systems, Institute of Automation, Chinese Academy of Sciences, Beijing 100190, China.; ^2^ School of Artificial Intelligence, University of Chinese Academy of Sciences, Beijing 100049, China.; ^3^ AMAP, Univ. Montpellier, CIRAD, CNRS, INRAE, IRD, Montpellier F-34398, France.

## Abstract

It is crucial to assess the impact of climate change on crop productivity and sustainability for the development of effective adaptation measures. Crop models are essential for quantifying this impact on crop yields. To better express crops’ intrinsic growth and development patterns and their plasticity under different environmental conditions, the functional–structural plant model (FSPM) “GreenLab” has been developed. GreenLab is an organ-level model that can describe the intrinsic growth and development patterns of plants based on mathematical expressions without considering the influence of environmental factors, and then simulate the growth and development of plants in expressing plant plasticity under different environmental conditions. Moreover, the distinctive feature of GreenLab lies in its ability to compute model source–sink parameters affecting biomass production and allocation based on measured plant data. Over the past two decades, the GreenLab model has undergone continuous development, incorporating novel modeling methods and techniques, including the dual-scale automaton, substructure methods, the inverse of source–sink parameters, crown analysis, organic series, potential structure, and parameter optimization techniques. This paper reviews the development history, the basic concepts, main theories, characteristics, and applications of the GreenLab model. Additionally, we introduce the software tools that implement the GreenLab model. Last, we discuss the perspectives and directions for the GreenLab model’s future development.

## Introduction

Food security is increasingly challenged worldwide due to climate change and the rapidly growing populations [1]. According to the World Population Prospects 2022 report released by the United Nations [2], the global population is expected to reach 9.7 billion by 2050, making it crucial to assess the impact of climate change on crop yields and explore effective strategies for future agricultural sustainability [3].

To tackle the challenges of climate change, two main strategies are generally taken. The first involves genetic improvements aiming at developing new crop varieties that are better adapted to changing climate conditions and more efficient in the use of resources. The second strategy involves adjustments to crop management practices. However, it is crucial to quantify and assess the impacts of both genetic improvement and management adjustments on crop production [3]. Furthermore, to develop effective adaptation measures, it is essential to estimate how climate change will affect crop productivity and sustainability [4].

The growth of crops is influenced by a combination of genetic factors (G), environment (E), and management measures (M) [5]. Crop models are specifically designed to simulate G × E × M interactions, which can assess the impact of climate change on crop production and identify appropriate adjustment measures to counteract any negative effects of climate change on yield [6]. As a result, crop models are considered a crucial tool for quantifying the impact of climate change on crop yields [5].

Crop models can be categorized into two types: (a) statistical models (SMs), also known as data-based models, and (b) process-based models (PBMs), also known as knowledge-based models. The former directly establishes the relationship between environmental inputs and yield outputs without concerning the inherent growth processes of the crops [7,8]. The latter, on the other hand, focuses on describing the essential physiological and physical processes that influence yield formation [9–11].

SMs primarily include empirical regression methods, machine learning (ML) algorithms, and deep learning (DL) algorithms [12]. Compared to PBMs, SMs demonstrate relatively higher predictive capability when sufficient training data are available. However, this also means that they heavily rely on the data [13]. Due to the highly complex nonlinear relationships between crop yield and input variables, issues like overfitting, prolonged training, and relatively fewer hidden layers restrict their ability to address nonlinear problems and predict crop yields over large areas [14,15]. PBMs simulate various processes in crops, such as photosynthesis and assimilate allocation. They primarily use the leaf area index (LAI) to predict biomass yield per unit of land area and consider the influence of environmental factors like light radiation, temperature, and management practices (irrigation, fertilization, etc.). Common PBMs include DSSAT (Decision Support System for Agrotechnology Transfer) [16], TomSim [17], STICS (Simulateur mulTIdisciplinaire pour les Cultures Standard) [18], and APSIM (Agricultural Production Systems sIMulator) [19].

PBMs often consider different types of organs, focusing on the total fruit weight, leaf weight, etc., making it difficult to describe the influence of changes in crop structure on yield. During their growth, plants undergo various adaptive responses in terms of intrinsic physiology and external morphology by regulating their characteristics, such as size, number, and growth volume, to minimize adverse effects of the environment. Therefore, to accurately predict crop yield, crop models need to consider the environmental effects on plant structure. To better express the intrinsic growth and development patterns of crops and their plasticity under different environmental conditions, functional–structural plant models (FSPMs) have been developed. This led to the development of organ-level FSPMs, which simulate crop morphological structure, biomass production and allocation, and the inherent relationships between them [20,21]. These models operate at a finer scale, providing more detailed simulations of crop growth. Representative models in this category include ALMIS [22], LIGNUM [23], L-Peach [24], and GreenLab [25]. FSPMs simulate the two basic processes of plant growth and development by combining physiological functions to represent three-dimensional (3D) plant structure [20], and link the joint effect of internal growth and external environment with plant architecture [26].

The GreenLab model is one of the FSPMs, taking into account the characteristics of the sequential production of similar organs and adopting the concept of a common pool and source–sink relationship. It models plant growth at the leaf element (organ) scale while maintaining compatibility with PBMs (at the population level). To build the GreenLab model, an architectural model was coupled with a growth model with source–sink functions of individual organs [27]. This framework formalized the organogenesis, photosynthesis, and morphogenesis processes of plants. Unlike PBMs, which model the sink and source processes at the whole plant level, GreenLab models these processes at the level of individual organs, each having its age according to its position within the plant structure [28]. This allows a more detailed and accurate simulation of plant growth and development.

Based on the plant automaton, in the GreenLab model, a recursive algorithm is used to calculate the number of organs produced at each time step, and the biomass saved in the common pool is distributed to different categories of organs based on their age, the relative sink strength, and organ numbers. This process is described by mathematical formulas, eliminating the need to simulate the biomass allocation of each organ individually, making it faster and requiring less time, which is one of the advantages of the GreenLab model. The distinctive feature of GreenLab lies in its ability to compute model source–sink parameters affecting biomass production and allocation based on measured plant data such as the weight of each plant organ. Additionally, the model’s effectiveness can be determined through simulation, which reduces the need for extensive data collection [29]. Based on generic botanical and eco-physiological knowledge, the model has been applied to study over a dozen crops, such as corn, wheat, rapeseed, cucumber, and tomato [30]. However, the GreenLab model simplifies the effects of environmental factors into a single environmental factor “*E*,” which cannot effectively simulate the effects of climate, soil, and management practices on crop yield.

This paper reviews the development course, the basic concepts, the main theories and characteristics, the applications of the GreenLab model, and the software tools. The “The Developmental History of the GreenLab Model” section presents the developmental history of the GreenLab model. The “The GreenLab Model” section introduces the concepts and assumptions used in the GreenLab model. The “Applications of the GreenLab Model” section presents the applications in crops and trees. The “Software” section presents the software tools implemented based on the GreenLab model. The “Perspectives” section gives the perspectives and future directions of the GreenLab model. The “Conclusions” section gives conclusions.

## The Developmental History of the GreenLab Model

The GreenLab model originated from the AMAP (botAny and Modelling of Plant Architecture and vegetation) modeling approach, inheriting botanical concepts from AMAPsim [31,32] such as physiological age (PA) and reiteration growth, as well as the source–sink concept from AMAPHydro [33]. The AMAP plant library is based on the architectural models proposed by Hallé et al. [34], which ensures that the simulation of plant development is faithful to botanical principles [35]. This feature laid the foundation for the application of the model in agronomy.

**Fig. 1.**
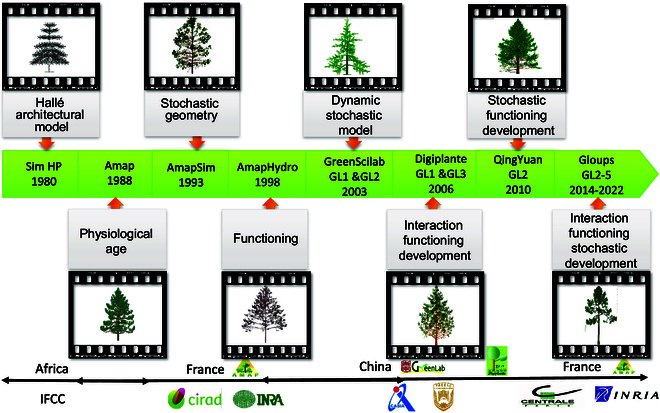
Developmental history of the GreenLab model. The GreenLab model originated from the AMAP modeling approach, inheriting botanical concepts from AMAPsim and AMAPHydro, which was developed within the collaborative efforts of the Sino-French Joint Laboratory (LIAMA) in 1998. The GreenLab model progressed through various iterations, including GL1 (deterministic model), GL2 (stochastic model), GL3 (deterministic model with feedback mechanisms), and eventually GL4 and GL5 (stochastic model with feedback mechanisms). Notably, GL5 represents a fine time-scale model for trees, which can simulate the growth and development of trees within a year.

To meet the need for growth process models, the simulations of biomass production and distribution were introduced into the AMAP models, and AMAPHydro was proposed, which uses water as the growth driver [33]. Since 1998, based on the Sino-French Joint Laboratory (LIAMA), the cooperation between the Institute of Automation, Chinese Academy of Sciences (CASIA) in China and the International Cooperation of Agronomy Research and Development Centre (CIRAD) in France was established, leading to the development of the organ-scale FSPM “GreenLab” (Chinese name Qingyuan), which inherits key concepts of the AMAP model series. Over the past 20 years, several institutes and universities have contributed to its development, including China Agricultural University (CAU), Chinese Academy of Forest (CAF) in China, and French National Research Institute for Digital Science and Technology (INRIA), Ecole Centrale Paris (ECP) in France, as shown in Fig. 1.

The architectural model in GreenLab is based on the concepts of AMAPSim [31] and can simulate the 23 botanical architectural models [34,36]. It is designed to simulate complex plant structures, including trees, by organizing the model according to PA and using only a limited number of parameters. This makes it easy for users to construct even very intricate plant structures. The functional model shares the hypothesis and concepts of PBMs, such as the common pool, the sink strength, and the sink variation. Therefore, the loop of plant growth is compatible with other PBMs.

GreenLab is a plant growth and development model that has been developed and refined over several levels of complexity. The initial model, GL1, is deterministic and relies on predefined rules for plant development [27]. The structure of plants is fixed and unable to adapt to internal or external factors [27,37], which limits its applicability to single-stemmed species, such as maize and sunflower. The GL2 model incorporates stochastic development and probabilistic bud outbreak to simulate the variation in plant structures [26,28,38], making it useful for assessing and comparing different cultivars or breeding programs aiming at developing plants with stable and reproducible behavior (low variation among individuals). This modeling strategy permits considering intra- and inter-individual variation and describing the statistical outputs of a population like mean and standard deviation rather than the results of individual plants. The GL3 model is deterministic, but the development is controlled by a state variable known as the supply-to-demand ratio [39], which creates a feedback loop between development and functioning that makes the model dynamics self-regulating. The GL4 model incorporates the feedback mechanism of supply-to-demand ratio as a determinant of the stochastic plant development processes, resulting in a decrease in the root mean squared error [40]. A more systematic parameterization methodology of GL4 has been described by Letort et al. [29]. The GL5 model is a stochastic model with interaction between the development and growth of trees at a finer temporal scale, which can simulate multiple growth flushes of trees within a single growing season [25], making it useful for simulating the growth and development of perennial plants that undergo multiple growth cycles. Overall, the GreenLab model is a valuable tool for simulating plant growth and development, and its different versions offer a range of options for researchers to choose depending on their specific needs and research questions.

## The GreenLab Model

### Concepts and assumptions

The GreenLab model is a dynamic model that uses discrete simulation to model the production and allocation of biomass at the level of individual plant organs. It combines both functional and structural descriptions of physiological processes, including phytomer-level structures, making it possible to study the model at both organ and stand levels.

The GreenLab model uses some important notions [25,26]: (a) temporal scale. Three distinct ages are used to characterize a meristem: chronological age (CA), which denotes the duration of organ/plant functioning since its creation; PA, which indicates its level of differentiation in the aging process; and ontogenic age (OA), which corresponds to the time of its creation within the plant structure. Chronological and ontogenic ages are measured in development cycles (DCs), which serve as the fundamental unit of duration that governs plant development. During each DC, a meristem can produce either a phytomer or a pause. The cycle is measured in thermal time rather than calendar time, as it regulates the overall development process. (b) Spatial scale. Three kinds of concepts are defined to describe the structure of plants: organic series, which is the dimension or weight of organs produced sequentially along an axis of development; cohorts are a set of organs of the same nature, created at the same time by the parallel functioning of meristems; and plant crowns (the combination of primary bearing axes and secondary ramified axes).

The model incorporates eco-physiological concepts from crop models such as thermal time, light use efficiency (LUE), water use efficiency (WUE), and a common pool, among others, as described by de Reffye et al. [25]. To combine plant organogenesis and plant photosynthesis, the GreenLab model uses plant architectural models defined by Hallé [34], duel-scale automaton theory [41], and substructure-based algorithm [42]. GreenLab uses a dual-scale automation approach to generate stochastic structures of plants, which integrates botanical knowledge, such as phytomers and growth units (GUs), to construct topological and morphological structures of plants through a graph-based interface [43,44]. For more complex applications involving trees or plantations, the GreenLab model employs a strategy of substructures [45] for efficient construction of plants and yields calculation based on organ production. Using the same temporal scale for development and growth, the model simulates plant growth behavior recurrently and can be applied to a range of plant types, from herbaceous plants to trees. While the basic structure of the model is generic, corresponding submodules can be introduced for different plant species.

**Fig. 2.**
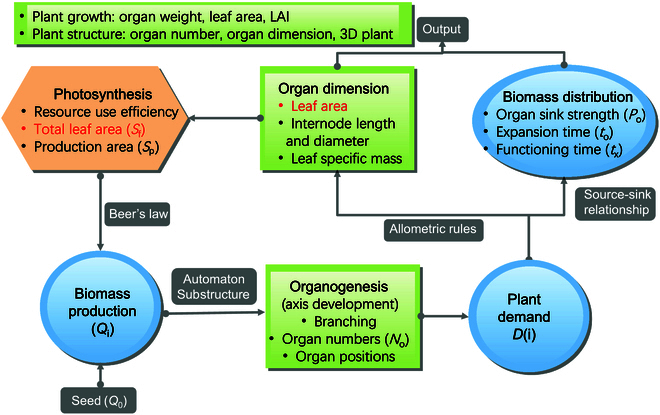
A diagram depicting the simulation process of the GreenLab model. The green rectangles represent the development, and the blue circles represent the growth. The model outputs and the computed model parameters are given. Biomass production can be computed through photosynthesis (in orange).

The GreenLab model employs compact mathematical equations with integrated parameters for plant growth simulation, allowing easy parameterization. Organs of the same features share the same function, without requiring a complex plant structure description that can be laborious. In the model, plants start from a seed, which provides the initial source, allocate biomass to existing organs based on the source–sink relationship, make photosynthesis according to functioning leaf area, and determine the number and size of organs at different stages. The model iteratively simulates plant growth cycle by cycle until it ceases (Fig. 2), providing a comprehensive understanding of biomass production and partitioning. At the end of the simulation, the plant’s architecture can be displayed in 2D or 3D format [29,46].

### Modeling plant development and growth

Plant morphogenesis arises from two main phenomena: development and growth. Development is facilitated by the functioning of meristems, which give rise to a plant structure comprising branched axes composed of phytomers in a series. On the other hand, growth is the process of biomass accumulation in the plant system through photosynthesis. This growth is regulated by source–sink relationships, where organs perform their functional roles. It ensures the increase in biomass of the organs formed during development.

In this review, we will not give detailed descriptions of modeling plant development and growth, which can be found in [25,30]. Instead, we will provide a concise overview of the important concepts used in the GreenLab model and highlight their roles.

#### A botanical automaton to simulate the development

A phytomer is a fundamental unit in plant structure, consisting of an internode that terminates in a node where organs like leaves, fruits, and axillary meristems are attached. Depending on whether the flowering occurs at the axis or the tip, it can be axial or terminal. To simulate the growth and development of plant structures, it is sufficient to describe the rules that govern the PA of newly produced phytomers. To accomplish this, a dual-scale automaton approach using graph-based notations [36] was developed. Structures can be simple or compound, depending on whether meristems function continuously or rhythmically. In the latter case, mainly in trees, the automaton is on a double scale, with two temporal scales being considered and meristems setting up the GUs. The micro-temporal cycle corresponds to the creation of a phytomer, while the macrocycle represents the construction of a GU. Structural factorization is highly efficient in designing, understanding, or simulating plant architecture models [45]. Over recent years, there has been significant progress in extending the formalism to encompass stochastic cases. This has been achieved by introducing probabilities to represent the likelihood of transition occurrences. Furthermore, this extended formalism has undergone validation across numerous plant species [25,26,29].

#### Modeling biomass production and partitioning

The GreenLab model, similar to other crop models, uses the concept of a common pool that stores synthesized biomass and distributes it to organ compartments [25]. The model uses a discretized beta law function to define the sink function [47], and the plant demand at a given age is calculated as the sum of the active sink organs. The number of phytomers produced by the botanical automaton determines the number of leaves, internodes, and fruits produced in each cycle.

To compute biomass production and partitioning, first the biomass supply is required. Like other crop models, it uses Beer–Lambert’s law to calculate biomass production per unit of cultivated area and per unit of time. Then, plant demand is calculated in each DC, and the biomass growth of an organ depends on the value of its sink and the ratio of the biomass supplied in the previous cycle to the current demand.

### Model parameter estimation

As the GreenLab model consists of two modules, one for organogenesis (development) and the other for organ growth, its key parameters can be categorized into two parts as well. The first set of parameters for plant development, which includes branching rate, growth rate, and mortality rate, is computed using the measured number of phytomers along the stem from the top to the bottom, a method known as plant crown analysis [48]. On the other hand, the second set of parameters for organ growth, which includes the source parameters, such as seed biomass (*Q*_0_), projected surface (*Sp*), and resistance coefficient (*r*, related to LUE or WUE), and the sink parameters, such as sink strengths (*P_o_*, *o* represents leaf, internode, fruit, etc.) and the variations of sink strengths (*B_o_*), are identified by fitting the measured organic series and controlling the functional model. As explained before, the description of each organic series contains all the necessary information for development and growth. Through adapted sampling within the organic series, effective targets for calibration of the source–sink parameters can be defined using experimental data. Organic series are constructed by sampling within the plant architecture, and measurements can be taken at multiple growth stages. Besides, some empirical parameters are needed, such as the expansion time and functioning time of organs, and leaf thickness, which can be obtained according to the observed data during the growth of plants.

The process of identifying parameters in the GreenLab model involves three steps: (1) measuring the architecture of the plant, including the number of leaves, internodes, fruits, and the number of phytomers within the stem and branches, thus to know the topological structure of the entire plant (to know the development parameters) and also the times of expansion and functioning of each type of organs; (2) constructing target data (organic series and/or compartment data) by measuring dry or fresh weights of each organ (leaves, internodes, fruits, etc.); (3) fitting the target data and estimating functional source–sink hidden parameters. The developmental parameters, such as growth and branching rates, are determined using the crown analysis method as mentioned above. Model calibration can be done by single fitting, where only one stage of data is used to fit the organs, or multi-fitting, which involves using data from multiple growth stages to fit the organic series [47].

**Fig. 3.**
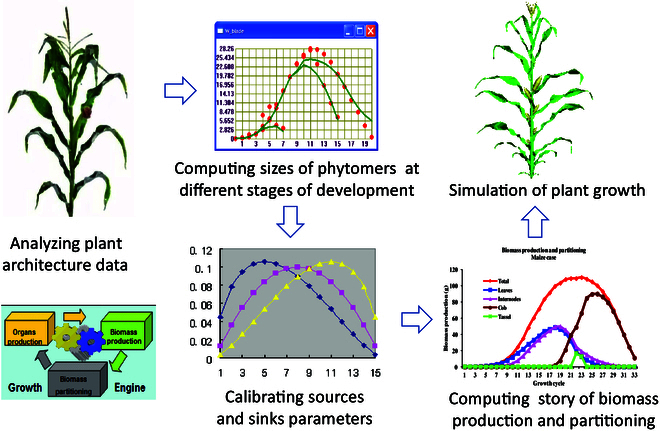
Parameter estimation framework for the GreenLab model. The GreenLab model can perform dynamic plant growth and development based on static plant architecture. By analyzing plant architecture data, GreenLab can compute the size and weight of organs at various stages. Subsequently, it estimates source and sink parameters from the measured data using the WLSM. This process enables GreenLab to describe the story of biomass production and partitioning within the plant. Finally, the model can display the plant in 2D or 3D.

As such, the GreenLab model can simulate the dynamic progression of plant growth and development while relying on a stationary plant architecture (Fig. 3). By analyzing plant architecture data, GreenLab can compute the size and weight of organs at different stages, and then estimate source and sink parameters using the weighted least square method (WLSM) with measured data. Calibration of the model on real plants is a crucial step for its application, but it is a time-consuming and tedious process. Therefore, GreenLab defines a uniform data sampling scheme to simplify the measurements [25,26].

## Applications of the GreenLab Model

Plant structures can be categorized into various architectural models based on different modes of meristem functioning (continuous/rhythmic/polycyclic, definite/indefinite), branching patterns (monopodial/sympodial), and flowering types (apical/lateral). These architectural models proposed by Hallé et al. [34] constitute a comprehensive set that covers all types of plants. By utilizing specific sampling strategies and appropriate parameter estimation methods (WLSM), plant system parameters can be identified. This approach enables the effective modeling of many herbaceous and woody plants, as they can be accommodated within these architectural models.

**Fig. 4.**
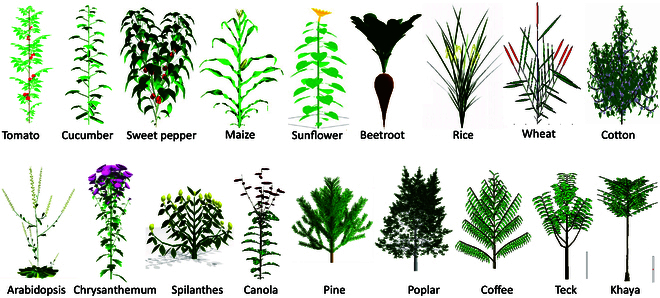
Plant species studied with the GreenLab model. The GreenLab model has demonstrated its versatility by successfully studying a wide range of plant species, including both temperate and tropical species with continuous growth and rhythmic growth. The development and growth parameters of the model have been effectively estimated for more than 20 plant species, including field crops, horticultural crops, herbaceous plants, and trees.

The GreenLab model has been successfully applied to both temperate and tropical species, covering continuous growth and rhythmic growth, and accounting for the stochastic effects of phytomer development, branching, and viability. The development and growth parameters of the model have been satisfactorily estimated using crown and organic series analysis [25] for over 20 plant species; the description and the characteristics of development and growth for the main species are listed in Table 1. Figure 4 shows the 3D visualization of plants for various species.

**Table 1.** Plant species studied with the GreenLab model and the descriptions of the corresponding models

Plants pecies	Plant description	Developmental and growth patterns	References
Field crops			
Maize	A single-stemmed plant, deterministic and continuous development. After an initial rosette stage, the stem lengthens and the development stops after about 20 phytomers (depending on varieties) following terminal flowering with the formation of a male flower.	Basic phytomer: an internode, a leaf made of a blade, and a sheath.Mask file: to note the position of the cob within the plant.Simple organic series: the blades, sheaths, internodes, and ears on the stem.	[47,49,50]
Sunflower	A single-stemmed plant, deterministic and continuous development.After an initial rosette stage, the stem lengthens and the development stops following terminal flowering (Capitulum).	Basic phytomer: an internode, a blade, and a petiole.Mask file: to note the position of the capitulum.Simple organic series: the blades, petioles, internodes, and caps on the stem.	[27,79,98]
Beetroot	A single-stemmed plant, deterministic and continuous development.	Basic phytomer: a leaf.Simple organic series: the rosette of leaves and the tape root on the stem.	[91]
Wheat	A branched plant, stochastic and continuous development, characterized by an initial rosette-like stage, followed by an elongation of the stem which ends in an ear. A variable number of secondary stems, called tillers, are issued at the same time as the main stem at the rosette stage. Remobilization of leaves needs to be considered.	Basic phytomer: an internode, a leaf, and a sheath.Crown analysis: the probabilities of growth, branching, and mortality were computed.Simple organic series: leaf, sheath, internode, ear on the main stem;Compound organic series: compartments of each type of organ for tillers.	[99]
Rice	A branched plant, stochastic and continuous development characterized by an initial rosette-like stage, followed by an elongation of the stalk ending in a spike. Tillers are issued at the same time as the main stem at the rosette stage. Remobilization of leaves needs to be considered.	Basic phytomer: an internode, a leaf, and a sheath.Mask file: to note the positions of the tillers and fruits within the plant.Simple organic series: blade, sheath, internode, and terminal spike for the stem and tillers.	[100,101]
Cotton	A branched plant, and the stems and vegetative branches of the plant have deterministic and continuous development. They have monopodial branching and bear fruiting branches with a sympodial structure.	Basic phytomer: an internode, a blade, and a petiole.Three PAs: the stem, its vegetative branches, and the fruiting branches.Mask file: to note the positions of the tillers and fruits within the plant.Simple organic series: blades, petioles, and internodes for the stem and branches.	[102–104]
Horticultural crops			
Tomato	A branched plant with a sympodial modular structure, but one of the branches is pruned, leaving a stem with a continuous development at the beginning and after a rhythmic development. The expansion of fruits has a delay.	Basic phytomer: an internode, a blade, a petiole, and a fruit.One PA: the stem, a single-stemmed plant was analyzed.Mask file: to note the position of the fruits.Simple organic series: blades, petioles, internodes, and fruits on the main stem.	[51,52,105,106]
Cucumber	A single-stemmed plant whose stem, in the shape of a vine, has continuous development and growth. The expansion of fruits has a delay.	Basic phytomer: an internode, a blade, a petiole, and a fruit.One PA: the stem, a single-stemmed plant was analyzed.Mask file: to note the position of the fruits.Simple organic series: blades, petioles, internodes, and fruits on the main stem.	[53,85,107]
Sweet pepper	A branched plant with stochastic and continuous development. After germination, the unbranched stem stops its development early because of an apical flowering that produces the first fruit. Two sympodial branches of equal vigor are born immediately under the last two phytomers of the stem. Their continuous development is limited to a few phytomers, after which branching becomes sympodial.	Basic phytomer: an internode, a blade, a petiole, and a fruit. Three PAs: main stem, branches, and twigs. Mask file: to note the position of the branches and fruits. Simple organic series: blades, petioles, internodes, and fruits on the main stem, branches, and twigs (12 organic series). The topological structure of a plant is simplified to facilitate measurements and target preparation.	[55,108,109]
Herbaceous plants with inflorescences			
Arabidopsis	A single-stemmed plant at the vegetative stage with deterministic and continuous development. The beginning of the growth for the *Arabidopsis* is in a rosette form. At the generative stage, branching inflorescence appears.	Basic phytomer: a leaf. One PA: the stem Simple organic series: leaves.	[58,59,93]
Canola	A branched plant with stochastic and continuous development. The beginning of the growth is in a rosette form. Following the emergence of the leaves, internodes of the main stem begin to elongate. At the growth end, the apical meristem of the stems transforms into an inflorescence. Flower emergence starts on the main inflorescence and develops basipetally to the lateral inflorescences, while the flowers within the inflorescence have an acropetal flowering sequence. Remobilization needs to be considered.	Basic phytomer: an internode, a leaf, and potentially a ramification. Two PAs: main stem and ramifications. Crown analysis: the development parameters rhythm ratio, branching rate, and growth rate are computed. Simple organic series: leaves, internodes, and fruits on the main stem;Compound organic series: the organs of the same type (leaves, internodes, fruits) are weighed separately for ramifications. Delay function: to simulate the basipetal pattern of raceme development.	[60–63]
Chrysanthemum	Chrysanthemum is a short-day plant with stochastic and continuous development, having a basipetal flowering sequence.	Basic phytomer: an internode, a leaf, and potentially a flowering branch.Three PAs: main stem and ramifications.Simple organic series: leaves, internodes, and fruits on the main stem and ramifications.Delay function: to simulate the basipetal pattern of raceme development.	[56,57,64]
Spilanthes	A branched plant with a stochastic and continuous development. The stem is short and the shoots spread. All axes end with a terminal flower. After a minimal rosette stage, the stem elongates and places a small number of phytomers, then the apical flowering stops development, and the preformed lateral branches begin to expand with acropetal development. If conditions allow, the preformed third-order branches begin their expansion with a basipetal development.	Basic phytomer: an internode, two leaves, and potentially two twigs.Three PAs: main stem, branches, and twigs.Crown analysis: the development parameters rhythm ratio, branching rate, and growth rate are computed.Simple organic series: leaves, internodes, and fruits on the main stem;Compound organic series: the organs of the same type of ramification are weighed separately by compartments (leaves, internodes, fruits) for ramifications.Delay function: to simulate the basipetal pattern of raceme development.	[40]
Trees			
Coffee	Coffee is a woody shrub plant with a stochastic and continuous development. The stem is orthotropic, and each phytomer has 2 leaves with potentially 2 plagiotropic branches. At the young stage, there is no mortality or flowering.	Basic phytomer: an internode and two leaves.Three PAs: main stem, branches, and twigs. Crown analysis: the development parameters rhythm ratio, branching rate, and growth rate are computed.Simple organic series: leaves, internodes on the main stem.Compound organic series: leaves, internodes, and fruits on the branches.	[29,110,111]
Pine	The pine tree is a conifer species with a stochastic and rhythmic development. Each year, the terminal meristems produce a new GU. The stochastic aspect only exists in the distribution of the number of branches per whorl.	Basic phytomer: an internode, a needle, and rings. The needles are considered as a single-leaf organ.Two PAs: main stem and branches. The growth cycle is the year.Crown analysis: the development parameters branching rate was computed.Simple organic series: the leaves and internodes of the stem and branches.	[38,65,66,112,113]
Teak	The teak is a tropical tree with stochastic and polycyclic development. It has polycyclic growth and branching. Its axes consist of a succession of GUs. Its flowering is terminal and generally occurs during the 5th year of growth. At the same time, the development of the axes, initially monopodial, becomes sympodial.	Basic phytomer: an internode, a leaf, and rings.GU: grouped according to their PA and CA and their ranks in the annual shoot.Four PAs: main stem and three orders of branches.Crown analysis: the development parameters branching rate was computed.Compound organic series: only the weights of the leaf and internode compartments per GU were measured.	[46]

To calibrate the model for each species, the process involves a few steps. First, the PA, CA, leaf thickness, expansion time, and functioning time of organs are collected based on observations during growth. Second, the development patterns of plants are analyzed and set. If a plant’s development follows a stochastic pattern, the developmental parameters are determined through two methods: a “mask” file is used to specify the positions of branches and fruits, essentially treating them as having deterministic development; alternatively, probabilities of growth, branching, and mortality are calculated using crown analysis method if the development is considered stochastic. Last, the source–sink parameters are estimated according to the measured weights of organs at different developmental stages, using either single or multi-stage fitting techniques.

### Modeling of field crops

For single-stemmed field crops, like maize, sunflower, and beetroot, their development patterns are treated as deterministic and continuous. The GreenLab model can directly compute model parameters using measured organ weights. However, for plants with branching like cotton, rice, and wheat, their developments are stochastic and continuous. To address this, a “mask” file has been used to document branch and fruit positions, transforming their stochastic growth into a deterministic form for cotton and rice. In the case of wheat, considered as a stochastic plant, initial developmental parameters such as branching rate, growth rate, and mortality are determined from observed data. Subsequently, the model parameters are fine-tuned by matching them with the measured organ weights. In the case of maize, it has been confirmed that the sink parameters remain consistent across different planting densities and growing seasons [49,50]. This stability in sink parameters holds significance for breeding efforts, as it allows for the identification of traits that remain unchanged despite fluctuations in the environment.

### Modeling of horticultural crops

For horticultural crops, such as tomato [51,52] and cucumber [53], their development follows deterministic and continuous patterns. The GreenLab model has been used to calibrate model parameters specifically for single-stemmed plants. In the case of branched plants like pepper [54], a mask file has been used to document fruit positions, effectively converting their stochastic growth into a more deterministic trajectory. These species’ yield formation is primarily influenced by the quantity and size of their fruits, underscoring the importance of accurately simulating fruit sets within these crop models. The GreenLab model has established a relationship between the internal source-to-reservoir ratio and fruit set through parameter inversion [51,55]. However, it is important to note that additional experiments are required to validate these predictions. In addition, studies have delved into understanding the variation in yield formation between different cucumber cultivars [53] and integration lines for tomatoes [52] using optimization algorithms. Nonetheless, the accuracy of these predictions needs validation through an extensive array of experiments.

### Modeling of herbaceous plants with inflorescences

The GreenLab model has specific adaptations for the growth and development of herbaceous plants with inflorescences [56]. In these plants, phytomers are created as a result of meristem activity, and organ expansion occurs following biomass accumulation. Herbaceous plants with inflorescences show great architectural variability and undergo two primary developmental stages: vegetative and reproductive. During the initial vegetative phase, phytomers are established according to the rules of botanical automaton, but their expansion does not occur immediately after creation. Instead, branches remain preformed until flowering initiation, with a delay influenced by creation time, branch type, and position within the topological structure generated by the development schedule. To model this delay in branch development, a delay function has been introduced [56,57]. The GreenLab model has been successfully applied to model herbaceous plants such as Arabidopsis [58,59], canola [60–63], chrysanthemum [56,57,64], and spilanthes [40].

### Modeling of trees

The GreenLab model can simulate growth rings and estimate their parameters through inverse estimation. This feature is unique to the model and distinguishes it from other functional structure models for trees, which have been extensively calibrated. Growth rings are first considered as an organ that competes with others, and the biomass allocated to them is then distributed to different internodes according to two different rules (e.g., Pressler law).

Apart from GreenLab, there are few FSPMs for trees that have been calibrated, meaning that the model parameters are determined based on tree measurement data to ensure that the calculated number of organs and biomass are consistent with the measured data. Representative model applications include a deterministic model for *Pinus tabulaeformis* [65], a stochastic model for *Pinus sylvestris* [38,66] (which can reflect the differences in individual structures and their impact on biomass), and a feedback model for *Fagus sylvatica* [67] (which establishes a relationship between the number of branches and the source–sink ratio).

The temporal scale of GreenLab for trees is generally annual, but more recently, a fine time-scale model has been developed for multiple pruning of trees [68], which is adapted to more complex cases where tree architectural development and biomass production occur simultaneously during long periods, where polycyclism, anticipated growth, or neoformation occurs. Periods of extension and rest can occur successively between GUs but also within GUs [68]. Indeed, GreenLab has been applied to several tree species, such as the simple structures like coffee and pine trees, and the complex structures like maple, teck [46], and khaya trees. Although we have not delved into the specifics here, it is worth mentioning that details regarding the results of maple and khaya trees are pending publication.

## Analyses of Model Behaviors

In addition to being calibrated for various species, the GreenLab model has also been applied to the optimization of maize yield [69] and tree wood production [70], irrigation [54], and plasticity of trees under different light conditions [71] and wind effect [72]. Furthermore, sensitivity analysis of model parameters has also been performed with the GreenLab model [73–75].

The GreenLab model can be integrated with other models or methods, such as the simulation study by Letort et al. [76], which incorporated genetics into the GreenLab model. This approach provides access to more fundamental traits for detecting quantitative trait loci (QTLs), offering potential tools for optimizing yield.

The GreenLab model can be integrated with ML methods to predict crop yield, like the study of Fan et al. [77]. They proposed a knowledge data-driven method (KDDM) by combining GreenLab and neural networks to predict tomato yield under different greenhouse environmental conditions. This KDDM has also been utilized for predicting crop yield under a controlled ecological life support system (CELSS) by computing the interaction between carbon, plants, and gases [78].

## Software

Since 2003, the software implementations of the GreenLab model have been made by different research teams that are available in different environments and have varying specifications. These implementations can support deterministic or stochastic simulations, and/or fitting based on the measured data, and feedback between growth and development.

GreenLab formalism is deployed in various programming languages and environments, including stand-alone simulation and calibration tools like Visualplant, Cornerfit, GreenScilab, Qingyuan, Gloups, StemGL, and XPlantGL. Table 2 provides a summary of these implementations and their specifications.

The initial software development focused on the generic simulation of plant architecture development using botanical automaton (Visualplant software). The second software, Cornerfit, focused on the growth simulation of single-stemmed plants, such as corn, sunflower, tomato, wheat, and beetroot using the WLSM [79]. The subsequent software, including GreenScilab, Digiplante, QingYuan, Gloups, and StemGL, synthesized the features of the first two software.

GreenScilab, the first complete open-source GreenLab software, offers a user manual, an interface for parameter input, a source–sink solver for organic series, and 3D output simulations. GreenScilab implements structural stochastic development, continuous and rhythmic growth, and source–sink production simulation. It takes advantage of substructure factorization for production and simulation and is particularly effective for plants with simple structures. The tool offers calibrated datasets on plants of agronomic interest based on field measurements [80,81].

**Table 2.** Software tools and their specifications are developed based on the GreenLab model

Software	Language	Main developer	Plant species	Features
Visualplant	C++	LIAMA, CASIA	Crops and trees	Deterministic, pure development;Simulation;
Cornerfit	C++	LIAMA, CASIA	Single-stemmed crops	Deterministic, single-stemmed;Simulation and fitting;
GreenScilab	Scilab	CASIA	Crops	Deterministic without feedback between growth and development; Simulation and fitting;
DigiPlante	C++	ECP	Crops and trees	Deterministic with feedback; Simulation and fitting;
dgpSDK libraries	C++	ECP	Crops and trees	Deterministic with feedback; Simulation and fitting;
QingYuan	C++	CASIA	Crops and trees	Deterministic with feedback;Simulation;
StemGL	Matlab or Octave	AMAP & Bioagressor, CIRAD	Single-stemmed crops	Deterministic without feedback;Simulation and fitting;
Gloups	Matlab	AMAP, CIRAD	Crops and trees	Stochastic and feedback; Simulation and fitting;
XPlantGL	Matlab	CASIA	Crops	Stochastic without feedback;Simulation and fitting;

StemGL is another open-source tool that focuses on single-stemmed plants and operates in both Matlab and Octave environments [82]. Gloups is the most advanced implementation, which enables the applications of both deterministic and stochastic models, but access to the tool requires a shared research project with specific terms of use [83]. For convenient applications, XPlantGL is developed only for crops; it can do simulation and fitting, including branching and stochastic plants. These implementations include a set of tools for parameterization, simulation, calibration, and optimization.

However, the specificity of scientific programming environments like Matlab or Scilab can limit scalability in terms of memory space and execution speed, prohibiting simulations of large numbers of phytomers that adult trees may present. To address this, versions designed in general-purpose languages like C, C++, and Java have been developed, such as Digiplante and QingYuan [84]. The Digiplante tool, piloted by the Digiplante team at École centrale Supélec, gives birth to the dgpSDK development environment that enables the simulation and visualization of 3D scenes from the GreenLab model. The QingYuan simulator, piloted by CASIA [84,85], is developing a cloud-based interface to facilitate model-based training and competition.

## Perspectives

### Combing with plant phenotyping for crop breeding

Plant breeding involves a repetitive cycle of assessing multiple generations before introducing an improved cultivar. The challenges posed by a growing global population and changing climate demand sustainable food production solutions. This process of developing unique and advanced cultivars with desirable traits for crop plants is time intensive and can span many years [86]. Traditional breeding methods typically take around 10 to 15 years, involving about 3 to 7 years for the development of initial lines, followed by 4 to 5 years of field testing, and then an additional 1 to 3 years for the official release of new cultivars. By shortening the generation cycle, it becomes feasible to significantly reduce this lengthy timeline [87].

Speed breeding [88] is an emerging technology aiming at shortening the breeding cycle, thus accelerating crop research programs through rapid generation advancement techniques. However, it is important to note that speed breeding is indeed one of the more expensive techniques. It requires specialized infrastructure to maintain controlled environments and specific equipment for precise trait selection, both of which come with substantial expenses [87]. One approach to overcome this challenge is to concentrate efforts on plant varieties that are particularly relevant to breeding goals and to integrate speed breeding with existing breeding techniques. Another avenue involves the use of crop models to support plant breeding efforts.

Crop models enable yield prediction and offer insights into the interplay between environmental factors and plant physiological processes, influencing crop growth and development [3]. Integrating diverse crop models offers a way to enhance our understanding of crop behavior and fully unlock the potential of these models. The GreenLab model considers the effects of plant structure, allowing it to leverage phenotype data such as crop height, leaf area, grain number, and physiological and photosynthetic attributes for calibrating model parameters. With the rapid advancement of equipment and technologies for acquiring crop phenotype information, a wide array of traits can now be comprehensively gathered at various scales, ranging from individual organs to entire plants [89]. This accelerated data collection simplifies the process of obtaining plant growth information compared to conventional methods. However, the challenge emerges in effectively analyzing the substantial volume of acquired phenotype data [90]. By merging environmental data with phenotyping information, the GreenLab model can simulate plant plasticity under varying environmental and management practices.

The GreenLab model is highly compatible with PBMs. For instance, Lemaire et al. [91] used the GreenLab model to calculate the projection area based on the weights of various plant organs, and the results were consistent with experimental findings. Feng [92] combined the GreenLab model with a differential statistical method to explore the transition of yield from individual corn plants to populations. Notably, the calculated results were in concordance with population-level measurements. These studies indicate that the GreenLab model can simulate the growth and development of various plant organs using organ-scale data. The model effectively bridges the gap between agronomic crop models and plant configuration models, contributing to a more comprehensive understanding of plant dynamics.

Furthermore, based on environmental and phenotyping data, the GreenLab model can construct simulation models for different crop varieties and lines. Its generic nature and stability across diverse environments enable it to simulate the growth and development of various varieties under different environmental conditions. Consequently, the model serves as a valuable tool for breeders, facilitating the selection of suitable varieties while reducing the need for extensive field experiments and associated costs.

### Combing with multi-level models for evaluating crop adaptation and yield prediction

The adaptation of crops to extreme climate conditions based on crop models needs to intuitively express the effects of fertilization, irrigation, and other cultivation measures on the individual growth of crops. Based on the research from the population scale to the individual scale, it is possible to quickly simulate changes in crop morphology under different management measures based on models without the need to adjust the size of each organ individually. Individual-scale plant models often include organ-scale submodels, which can be further combined with genetic models at the micro-scale. As studied by Letort et al. [76], the GreenLab model was used to link growth model parameters to QTL. Virtual genes and virtual chromosomes were defined to build a simple genetic model that drove the settings of the species-specific parameters of the model. A genetic algorithm was implemented to define the ideotype for yield maximization based on the model parameters and the associated allelic combination. Combining the GreenLab model with micro-scale (genetic and cellular scales) models can simulate crop growth and development processes under different genetic combinations and predict yields. Chew [93] developed a multiscale model for *Arabidopsis* rosette growth by integrating four existing models. This integration enabled the connection of genetic regulation and biochemical dynamics with processes occurring at the organ and whole-plant levels. This approach proved to be invaluable in understanding how the interplay between internal genetic factors and external environmental influences impacts the growth of *Arabidopsis*. Furthermore, the GreenLab model can also be combined with PBMs on a population scale, such as DSSAT or APSIM [94]. Individual-scale models can serve as a bridge, linking research efforts conducted across different scales. This cross-scale integration significantly facilitates more extensive model-driven research.

The integration of models operating at different spatial scales allows a comprehensive simulation of the intricate interplay between crop genetic traits, management practices, and environmental conditions, as well as the dynamic process of growth and development across various plant organs within an individual, facilitated by a profound comprehension of plant processes. Furthermore, this integration allows for a detailed description of yield composition and permits a 3D visualization of crops, which can then be compared with phenotype information. This fusion of different scale models not only offers valuable insights into plant breeding and phenotype characterization but also serves as a resource for optimizing crop systems.

### Combing with AI methods to produce simulated data

With the increasing adoption of sensor and communication technologies in agriculture, ML has emerged as a valuable tool for predicting yield and phenotyping. The GreenLab model stands to benefit from integration with artificial intelligence (AI) algorithms, such as the previously mentioned KDDM [77,78]. By leveraging sensor data, the KDDM simplifies model parameterization and improves yield prediction accuracy.

ML algorithms need abundant and high-quality data to enhance their performance. However, it is crucial to recognize that the quality and relevance of the data are as important as the quantity itself. The data used must accurately reflect the problem that needs to be addressed. Careful consideration of data collection, preprocessing, and augmentation techniques can maximize the use of available data, even when collecting a substantial amount of data is difficult. Simulation models based on mechanical principles can play an important role in generating additional data for refining training models, especially in situations where obtaining a significant volume of real-world data proves difficult.

Furthermore, the integration of AI algorithms with the GreenLab model or other crop models holds the promise of extending these models to broader scales, ranging from farm level to regional and even global level. An illustration of this potential is found in the work of Jeong et al. [95], who introduced a method for predicting rice yield at the pixel level by combining crop models with DL models like long short-term memory (LSTM) and 1D convolutional neural networks (1D-CNNs). Feng et al. [96] integrated the APSIM with regression models such as random forest or multivariate linear regression to dynamically predict wheat yield in southeastern Australia. Chen and Tao [97] combined remote sensing-derived leaf area index (LAI), weather predictions, and physiological crop models to forecast winter wheat yield over several years in the North China Plain. This convergence of AI algorithms with crop models bears tremendous potential to expand the boundaries of crop modeling, enabling simulations across various scales and domains.

## Conclusions

Over the past two decades, the GreenLab model has undergone continuous development, incorporating novel modeling methods and techniques, including the dual-scale automaton [41], substructure methods [42], the inverse of source–sink parameters [79], crown analysis [48], organic series and potential structure [26,29], and parameter optimization techniques [52,69,72]. Moreover, studies have explored the quantitative relationships between model parameters and genetic factors [76], the integration with the ML method [77], and the link with APSIM [94].

Functioning at the organ scale, the GreenLab model excels in simulating the growth and development of individual plant organs. It boasts the advantage of accounting for the feedback effects of structure on crop growth while also allowing parameter calibration using measured data. This flexibility makes it compatible with micro-scale genetic models [76] and photosynthesis models [71], leading to a more profound understanding of how genetic and physiological processes interact to influence plant growth and development [93].

Additionally, the GreenLab model can be combined with population-scale process models, allowing for a more comprehensive analysis of plant populations within different environmental conditions [94]. By bridging the gap between different scales of modeling, the GreenLab model enables researchers to investigate the impact of environmental factors and management practices on the productivity and sustainability of plant populations at various levels. In the era of phenomics, where substantial crop growth data can be acquired, the calibration of the GreenLab model for various species and environments becomes even more viable. This adaptability positions the GreenLab model as a crucial bridge connecting genetic and physiological aspects, thereby providing a wider range of research possibilities.

## Data Availability

There are no data used in this manuscript.
